# A Preliminary Study of Proinflammatory Cytokines and Depression Following West Nile Virus Infection

**DOI:** 10.3390/pathogens11060650

**Published:** 2022-06-04

**Authors:** Allison Lino, Timothy A. Erickson, Melissa S. Nolan, Kristy O. Murray, Shannon E. Ronca

**Affiliations:** 1 Department of Pediatrics, Section Tropical Medicine, Baylor College of Medicine, Houston, TX 77030, USA; allison.lino@bcm.edu (A.L.); terickso@bcm.edu (T.A.E.); 2 Arnold School of Public Health, University of South Carolina, Columbia, SC 29208, USA; msnolan@mailbox.sc.edu

**Keywords:** West Nile virus, depression, cytokine, chemokine, proinflammatory, flavivirus, CES-D

## Abstract

West Nile virus (WNV) is a neurotropic flavivirus that can cause acute febrile illness leading to neuroinvasive disease. Depression is a well-described outcome following infection, but the underlying pathogenic mechanisms are unknown. Proinflammatory cytokines play important roles in WNV infection, but their role in depression post-WNV remains unstudied. This research aimed to retrospectively evaluate associations between proinflammatory cytokines and new onset depression in a WNV cohort. Participants with asymptomatic WNV infection were significantly less likely to report new onset depression when compared to those with symptomatic disease. Participants with encephalitis and obesity were significantly more likely to report new onset depression post-infection. Based on univariate analysis of 15 antiviral or proinflammatory cytokines, depression was associated with elevated MCP-1 and decreased TNFα, whereas G-CSF was significantly elevated in those with a history of neuroinvasive WNV. However, no cytokines were statistically significant after adjusting for multiple comparisons using the Bonferroni method. While symptomatic WNV infection, encephalitis, and obesity were associated with new onset depression following infection, the role of proinflammatory cytokines requires additional studies. Further research involving paired acute-convalescent samples, larger sample sizes, and additional data points would provide additional insight into the impact of the inflammatory response on WNV-mediated depression.

## 1. Introduction

West Nile virus (WNV), a neurotropic flavivirus transmitted by the bite of an infected mosquito, emerged as a public health concern in North America following its introduction to the United States in 1999 [1,2,3,4]. WNV is propagated by its enzootic cycle, in which infected mosquitoes feed on key avian species, who subsequently develop high-titer viremia [5]. Once infected with an amplified viral load, mosquitoes can transmit disease to other birds, horses, and humans [6,7]. Although *Culex* spp. mosquito transmission accounts for most WNV infections, transmission has been documented via blood or plasma transfusions, organ transplants, intrauterine contact with the fetus, human breast milk consumption, percutaneously in a lab setting, and conjunctival exposure in occupational settings [8,9,10,11,12,13]. WNV is a leading cause of autochthonous arboviral disease and neuroinvasive arboviral disease in the United States [3,4,14,15]. The virus is a single-stranded, positive-sense RNA virus surrounded by a lipid created by the host cell’s endoplasmic reticulum thus contributing to the difficulty in developing an antiviral treatment without potentially damaging the host [4,16,17].

Approximately 80% of those infected with WNV are asymptomatic or subclinical [1], but those with symptoms experience an acute nonspecific systemic febrile illness termed West Nile fever (WNF) [3,16,18]. Some may progress to severe infections and develop neuroinvasive syndromes such as encephalitis, meningitis, and/or acute flaccid paralysis, termed West Nile neuroinvasive disease (WNND). Risk factors associated with the development of WNND include hypertension, immunosuppression, cardiovascular disease, renal insufficiency, tremors, among others [19,20]. Long-term sequelae are common in WNND patients and may last for years after infection.

A cohort study following WNV-positive participants from 2002 to 2004 described that 31% of participants developed new onset depression, of which 75% met the Center for Epidemiologic Studies Depression (CES-D) definition for mild-to-severe depression [21]. An extension of this study followed the same cohort between 2003 and 2010, noting that 39% of participants self-reported experiencing new-onset depression, whereas 35% met the CES-D definition for clinical depression. Depression in these participants was described up to 8 years post-infection, providing additional evidence that depression is a long-term complication of WNV illness [22]. Other studies have also identified depression as an outcome of WNV illness, but none are known to address WNV-induced inflammation in the CNS as an effector for depression [23,24,25,26].

Although the mechanisms are not well understood, various studies have described high concentrations of immune signaling molecules among patient populations with depression [27,28,29]. These include cytokines, which are small, locally secreted proteins that modulate cell activity and regulate interactions between cells of the immune system [30,31]. Cytokines are involved in the regulation of immune responses, embryogenesis, inflammation, and wound healing, among others [30,31]. Cytokines involved in inflammation, called proinflammatory cytokines, can stimulate, recruit, or proliferate immune cells, causing acute inflammation and provoking fever [32]. Proinflammatory cytokines, particularly IL-6, IFN-γ, IL-1β, and TNF, also play a role in the development and maintenance of depressive illnesses, even partaking in treatment resistance. These cytokines are thought to modulate hippocampal neurogenesis, resulting in detrimental effects on memory and cognition, as well as promoting depression [27,28,29]. Importantly, these cytokines have altered expression in WNV patients; therefore, we hypothesize that cytokines upregulated by WNV infection may contribute to a depressive behavioral state. With evidence supporting a link between WNV infection and depression, we aimed to perform a preliminary analysis of cytokine variations in WNV participants with and without depression.

## 2. Materials and Methods

### 2.1. Study Design and Setting

This study analyzed previously collected cytokine data from a prospective WNV cohort established in 2002 in Houston, Texas [21,22,33]. Participants were originally identified through blood screening at local blood donation centers or local health department surveillance from 2002–2013. Original study protocols and procedures were approved by the University of Texas Health Science Center and then by the Baylor College of Medicine institutional review boards.

### 2.2. Study Population and Data Collection

The study population consists of 53 adults and adolescents infected with WNV who consented to participate in the study between 1 January 2002 and 31 December 2013.

A questionnaire was administered to each participant either in person, by email, or over the phone. The questionnaire captured self-reported signs and symptoms, onset, demographics, and previous medical history. Subsequent “follow-up” questionnaires were collected following participants’ illness and into recovery, if applicable. Self-reported depression was recorded by asking the participant if they have “had problems with depression since WNV”. History of depression was recorded by asking participants if they had been told by a doctor that they had depression or mental illness, and if so, what type, as well as asking if they had “problems with depression before [they] became ill”.

In addition to the questionnaire, the CES-D was used to evaluate clinical depression [34]. This assessment was composed of 20 questions pertaining to depressive moods and behavioral changes experienced during the prior week. Scores less than 15 indicate that the patient is not experiencing depression. Scores 15 or higher indicate the patient is experiencing clinical depression, with 15–21 indicating mild-to-moderate depression and greater than 22 indicating major depression [21].

**Figure 1:**
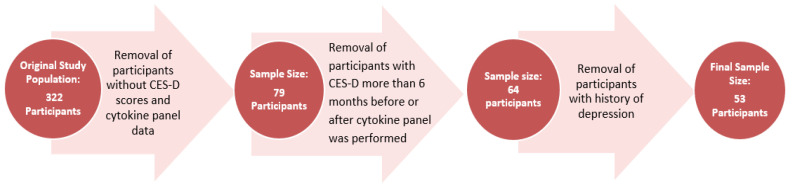
Participant Selection Process.

Participants with a history of depression prior to WNV infection were excluded from the dataset. The final study population yielded 53 participants who were tested for cytokines and had a CES-D assessment collected within 6 months of the cytokine panel in 2011 (Figure 1).

Blood samples for the cytokine analysis were collected on 89 randomly selected participants enrolled between 1 January 2002, through 31 December 2007, and subsequently tested via a Luminex cytokine panel. The cytokine data consist of a panel of 15 antiviral or proinflammatory cytokines and growth factors performed using a Luminex IS 100 platform (Austin, TX, USA) with Miliplex Analyst software. The following cytokines were included in the analysis: granulocyte-colony stimulating factor (G-CSF), interferon alpha 2 (IFN-α2), Interferon gamma (IFN-γ), interleukin 1 alpha (IL-1α), interleukin1 beta (L-I1β), interleukin 6 (IL-6), interleukin 8 (IL-8), interleukin 12p40 (IL-12p40), interleukin 17 alpha (IL-17α), interferon gamma-induced protein 10 (IP-10), monocyte chemoattractant protein-1 (MCP1), macrophage inflammatory protein 1alpha (MIP1α), macrophage inflammatory protein 1 beta (MIP1β), tumor necrosis factor alpha (TNFα), and tumor necrosis factor beta (TNFβ).

### 2.3. Participant Characteristics

Self-reported depression was defined as answering yes to the question “Do you feel like you have had problems with depression since your WNV infection?” Clinical evidence of depression was defined as having a CES-D score greater than 15. We used descriptive statistics to determine the prevalence of both these variables among study participants. We then performed a univariate analysis using logistic regression to examine demographics (sex, age, race, ethnicity), years post-infection, severity of infection (neuroinvasive vs. non-neuroinvasive), diagnosis (asymptomatic, West Nile fever, meningitis, encephalitis), depression risk factors (alcohol consumption, obesity, diabetes, hypertension, cardiovascular disease) and other variables of interest (depression medicine intake, counseling for depression) on the dependent variable of depression. Variables with p-values less than 0.05 on univariate analysis were considered statistically significant. Odds ratios (OR) and 95% confidence intervals (CI) for odds were also recorded. The same approach was used for “clinical evidence of depression.” Multivariate models were also explored adjusting these variables for age, sex, and race.

### 2.4. Depression and Cytokine Analysis

Cytokine data for the 15 antiviral or proinflammatory cytokines, and growth factors were analyzed as continuous variables with the outcome variables of self-reported depression, clinical evidence of depression, and history of WNND. A preliminary univariate analysis using the Kruskal–Wallis one-way ANOVA on ranks was performed, then the Bonferroni correction was applied to adjust the statistical significance threshold to account for 15 multiple comparisons. This lowered the p-value threshold for significance from 0.05 to 0.003. All data were analyzed using the NCSS 2020 Statistical Software (NCSS, LLC., Kaysville, UT, USA).

## 3. Results

### 3.1. Descriptive Statistics

The final study population consisted of 53 participants; more than half (*n* = 31; 59%) reported new onset depression since their WNV infection, and 21 (40%) had clinical evidence of depression based on their CES-D scores, including one who did not report new onset depression (Table 1). The majority of participants were male (53%) and non-Hispanic, white (37%). While not statistically significant, a higher proportion of women reported new onset depression since WNV infection (52%) and had CES-D scores of 15 or greater (57%) (Table 1).

### 3.2. Participant Characteristics

Preliminary univariate logistic regression for self-reported depression found that participants with asymptomatic WNV infection (OR = 0.148, 95% CI = 0.027 to 0.801; *p* = 0.027) were significantly less likely to self-report depression when compared to those with symptomatic disease, suggesting that asymptomatic infection is protective against depression. Logistic regression found that participants that take medication for depression (OR = 14.85, CI = 2.79 to 79.06; *p* = 0.002) and those that receive counseling for depression (OR = 11.20, CI = 1.23 to 101.89; *p* = 0.032) were significantly more likely to have clinical evidence of depression compared with those who reported not taking medicine or counseling for depression (Table 1).

After adjusting for age, sex, and race, the protective association between asymptomatic WNV infection (AOR = 0.13, 95% CI = 0.02 to 0.74; *p* = 0.021) and depression remained. Additionally, participants with WNND (AOR = 4.07, 95% CI = 1.09 to 15.18; *p* = 0.037), specifically encephalitis (AOR = 6.56, 95% CI = 1.38 to 31.28; *p* = 0.018), and obesity (AOR = 5.59, 95% CI = 1.21 to 25.75; *p* = 0.027) were significantly more likely to self-report depression (Table 2).

### 3.3. Cytokine Panel

All 53 participants included in the cytokine analysis provided self-reported depression information and had CES-D measures taken within 6 months of the cytokine measurements. Median and standard errors for the cytokine measurements were concentrations measured in picograms per milliliter (pg/mL) and are found in Table 3. Median and standard errors for participants without depression are included in Supplementary Materials Table S1. Univariate analysis using the Kruskal–Wallis one-way ANOVA on ranks yielded one conventionally statistically significant variable (MCP-1; *p* = 0.027) for self-reported depression, one conventionally significant variable (TNF-α; *p* = 0.013) for clinical evidence of depression, and one conventionally significant variable (G-CSF; *p* = 0.030) for history of WNND. Applying the necessary Bonferroni correction for 15 comparisons lowered the threshold of significance to a *p*-value of 0.003; therefore, no cytokine variables remained statistically significant.

## 4. Discussion

This study sought to provide a preliminary examination of how the inflammatory response post-WNV infection plays a role in the development of new-onset depression. While not considered statistically significant following the Bonferroni corrections, MCP-1, TNF- α, and depression had potential links upon univariate analysis and should be considered candidates for evaluation in future larger studies. Notably, MCP-1 and TNF-α are proinflammatory cytokines that have been identified in acute WNV disease [35,36]. MCP-1, in particular, is a proinflammatory chemokine produced by monocytes and macrophages that is involved in regulating the migration and infiltration of immune cells, as well as fibrosis and collagen tissue remodeling [36,37]. MCP-1, also known as chemokine C-C motif ligand-2, has been identified as present in all areas of the brain in patients with WNV [36] and has been shown to increase through the course of WNV infection [38]. MCP-1 has also been linked to depressive disorders, substantially increased during depressed states among those with bipolar disorder, [39] and was shown to decrease upon treatment with fluoxetine in patients with major depressive disorder [40]. This suggests MCP-1 elevations during WNV infection are a plausible player in WNV-induced depression and require additional study. Similarly, the proinflammatory cytokine TNF-α is associated with neuroinflammation, and other studies have identified its upregulation during acute WNV infection [41]. Similar to MCP-1, TNF-α is also associated with major depressive disorder, showing significantly higher levels when compared to control groups at baseline and decreasing levels upon antidepressant treatment [42]. These findings warrant further investigation, preferably with a larger sample size of patients compared to age-matched, WNV-negative participants. Previous research also highlights the importance of other immune modulators during WNV infection, notably including proinflammatory compliment C3 and C3aR, which are suggested to mediate hippocampal presynaptic terminal loss and spatial learning defects post-WNND recovery [43], and should be included in future studies.

After adjusting for age, sex, and race, we found self-reported depression to be associated with neuroinvasive disease, specifically encephalitis, and obesity. These findings suggest that symptomatic and severe WNV infections may be associated with self-reported depression and that other comorbid conditions could be contributing factors. Although the mechanisms are unclear, this is consistent with previous findings regarding increased depression among those with obesity [44,45]. The potential link between obesity and WNV infection may be of interest considering WN encephalitis could increase risk for obesity via higher neurocognitive control variability and lower physical quality of life [46]. Similarly, there may be a higher risk of obesity with other inflammatory conditions aside from WNV. Potential pathways for obesity and inflammation include inflammation induced by oxidative stress, physical fat cell rupture, and changes in microbiota resulting from diets high in fatty acids [47]. Future studies are necessary to evaluate these findings. WNND, which may present as encephalitis, meningitis, and/or acute flaccid paralysis, is associated with higher mortality rates, especially among older adults [3,19]. This higher risk among older adults coincides with study participants’ median age (51 years old), which could account for the larger proportion of neuroinvasive disease compared with febrile and asymptomatic disease. The development of depression after encephalitis has been observed in WNV and other central nervous system infections (i.e., St. Louis encephalitis virus and SARS-CoV-2) and idiopathic or autoimmune encephalitis (i.e., encephalitis lethargica, limbic encephalitis, and anti-NMDA receptor encephalitis) [48,49,50,51]. Additionally, upregulation of immune modulators such as IL-1β, IL-6, IL-8, IFN-α, IFN-γ, or TNF-α have been observed in other arboviral illnesses such as Zika, Toscana virus meningitis, and Japanese encephalitis virus [52,53,54], resulting in an inflammatory response that can trigger flu-like symptoms and more rarely, encephalitis [53]. The relationship between these cytokines and depression in these illnesses should be of interest for future studies.

Some limitations of this study must be noted. The most substantive limitation is the small sample size. No univariate comparisons were significant after correction for multiple comparisons. The Bonferroni method employed has been noted as overly conservative [55,56,57,58], but the limitation of our small sample size must be considered when evaluating this study. An additional limitation is the use of self-reported depression data, which could lead to information bias with participants potentially downplaying experiences, exaggerating experiences, or recalling events incorrectly. The CES-D depression scale also poses important considerations. Previous studies note that some CES-D items, such as item number 17 (“I had crying spells”) and item 11 (“My sleep was restless”), are susceptible to differences in interpretation and could differ between sex or culture [59]. Another potential limitation is related to selection bias, as the size of the study population, as well as the length and quality of follow-up measures, were limited by active participation in the study. In addition, this limited the diversity of the sample population, as the majority of participants were Caucasian, and the few from other races were not enough to determine the influence of race on WNV-mediated depression. Furthermore, the development of depression in WNND patients is likely influenced by the new disabilities and life-altering adjustments that occur as a result of neurologic sequelae. Therefore, further research is needed to determine the relationship between these factors and depression. Lastly, our study was unable to evaluate some immune markers and cytokines previously linked to WNV infection such as IL-2, IL-4, IFN-B, TLR-3, IL-11, and IL-18 [4,32,35], as well as some previously linked to depression such as IL-18 and NFκB [27,60]. Including these in future studies will be critical.

Overall, these results are from a small sample but are suggestive of a protective effect of asymptomatic infection on self-reported depression. Those with asymptomatic infection appeared less likely to develop new-onset depression when compared to symptomatic individuals, especially those with more severe neuroinvasive infections. This is biologically relevant as it may support that symptomatic WNV infection modulates physical responses, perhaps via the inflammatory response within the central nervous system, resulting in the development of depression as a long-term complication of disease. However, our findings did not specifically pinpoint the inflammatory pathways potentially responsible; therefore, additional research is necessary to determine causality. Future studies could consider incorporating additional depression instruments such as Beck’s Depression assessment and Social Problem-Solving Inventory—Revised (SPSI-RTM), although SPSI-RTM focuses on more than depression [61].

## 5. Conclusions

This research suggests symptomatic WNV infection, specifically neuroinvasive infection, may correlate with new-onset depression and suggests an association between obesity and depression after infection. These data highlight the need for more research about depression following WNV infection, particularly to evaluate the role of the immune system. Further study of the role of cytokines in WNV-associated depression is needed, preferably across multiple cohorts in a collaborative effort.

## Figures and Tables

**Table 1:** Univariate relationship of demographics and depression among all WNV cohort participants with no history of depression prior to West Nile virus (WNV) infection.

	All Participants (*n* = 53)	Self-Reported New Onset Depression Following WNV (*n* = 31)			Clinical Evidence of Depression with CES-D Scores ≥ 15 (*n* = 21)		
			*p*-Value ^†^	Odds Ratio (95% CI)		*p*-Value ^†^	Odds Ratio (95% CI)
Patient Demographics							
Sex, *n* (%)							
Male	28 (52.8%)	15 (48.4%)	0.443	0.65 (0.22, 1.96)	9 (42.9%)	0.241	0.51 (0.17, 1.57)
Age at onset, median (range)	50.7 (10–79)	49.9 (24–79)	0.823	1.00 (0.97, 1.04)	48.0 (24–79)	0.491	0.99 (0.95, 1.02)
Race, *n* (%)							
Caucasian	46 (86.8%)	29 (93.5%)	0.103	4.26 (0.74, 24.44)	19 (90.5%)	0.525	1.76 (0.31, 10.04)
African American	4 (7.5%)	1 (3.2%)	0.192	0.21 (0.02, 2.18)	1 (4.8%)	0.541	0.48 (0.05, 4.99)
Asian ^‡^	1 (1.9%)	0 (0.0%)	undefined	undefined	1 (4.8%)	undefined	undefined
Ethnicity, *n* (%)							
Hispanic	4 (7.5%)	3 (9.7%)	0.496	2.25 (0.22, 23.19)	1 (4.8%)	0.541	0.48 (0.05, 4.99)
Diagnosis, *n* (%)							
WNND	29 (54.7%)	20 (64.5%)	0.092	2.63 (0.85, 8.08)	12 (57.1%)	0.774	1.18 (0.39, 3.57)
Encephalitis	20 (37.7%)	15 (48.4%)	0.063	3.19 (0.94, 10.81)	7 (33.3%)	0.593	0.73 (0.23, 2.31)
WNF	15 (28.30%)	9 (29.0%)	0.889	1.09 (0.32, 3.69)	7 (33.3%)	0.511	1.50 (0.45, 5.03)
Asymptomatic	9 (17.0%)	2 (6.5%)	0.027 *	0.15 (0.03, 0.80)	2 (9.5%)	0.254	0.38 (0.07, 2.02)
Depression Risk Factors, *n* (%)							
Current Alcohol Consumption	29 (54.7%)	17 (54.8%)	0.455	1.65 (0.44, 6.17)	13 (61.9%)	0.188	2.71 (0.61, 11.94)
Obesity	17 (32.1%)	13 (41.9%)	0.053	3.68 (0.99, 13.77)	6 (28.6%)	0.879	0.91 (0.27, 3.10)
Diabetes	11 (20.8%)	9 (29.0%)	0.094	4.09 (0.79, 21.25)	6 (28.6%)	0.261	2.16 (0.56, 8.28)
Hypertension	19 (35.8%)	10 (32.3%)	0.683	0.78 (0.25, 2.51)	6 (28.6%)	0.494	0.65 (0.19, 2.21)
Heart Disease	6 (11.3%)	5 (16.1%)	0.183	4.55 (0.49, 42.31)	3 (14.3%)	0.503	1.80 (0.32, 10.06)
Other, *n* (%)							
Depression medicine	13 (24.5%)	13 (41.9%)	undefined	undefined	11 (52.4%)	0.002 *	14.85 (2.79, 79.06)
Depression counseling	7 (13.2%)	7 (22.6%)	undefined	undefined	6 (28.6%)	0.032 *	11.20 (1.23, 101.89)
Years Post-infection, median (std. error)	7.0 (0.267)	7.0 (0.376)	0.881	0.98 (0.74, 1.30)	7.0 (6.286)	0.610	0.93 (0.70, 1.24)

^†^ Wald *p*-values obtained by using univariate logistic regression; ^‡^ We compared Caucasian, African American, and Asian, respectively, to all other races. Measure limited by study sample size. * Statistically significant (*p*-value < 0.05).

**Table 2:** Multivariable adjusted logistic regression of disease factors on self-reported new onset depression.

	Self-Reported New Onset Depression Following WNV Infection		
	Adjusted Odds Ratio ^‡^	95% CI	*p*-Value ^†^
WNND	4.07	1.09, 15.18	0.037 *
Encephalitis	6.56	1.38, 31.28	0.018 *
WNF	0.88	0.22, 3.53	0.860
Asymptomatic	0.13	0.02, 0.74	0.021 *
Current Alcohol Consumption	1.56	0.39, 6.29	0.530
Obesity	5.59	1.21, 25.75	0.027 *
Diabetes	4.94	0.76, 32.22	0.095
Hypertension	0.92	0.25, 3.41	0.897
Heart disease	6.37	0.59, 68.77	0.127
Years post-infection	0.98	0.73, 1.32	0.910

^†^ Wald *p*-values obtained by using univariate logistic regression; ^‡^ Adjusted for age, sex, and race; * Statistically significant (*p*-value < 0.05).

**Table 3:** Cytokine concentrations in participants with self-reported depression, clinical depression, and history of WNND.

	Self-Reported Depression since WNV Infection (*n* = 31)		Clinical Evidence of Depression with CES-D Scores ≥ 15 (*n* = 21)		History of WNND (*n* = 29)	
	Median (Std. Error) **	*p*-Value ^†^	Median (Std. Error) **	*p*-Value ^†^	Median (Std. Error) **	*p*-Value ^†^
G-CSF	32.59 (5.402)	0.671	32.59 (6.570)	0.326	37.92 (16.542)	0.030 *
IL12p40	15.96 (10.576)	0.483	18.09 (12.533)	0.819	18.09 (15.368)	0.397
IL17α	2.32 (10.249)	0.117	2.32 (14.932)	0.938	2.32 (40.129)	0.481
IL1α	17.96 (26.225)	0.942	29.29 (37.858)	0.180	18.80 (14.700)	0.768
IL1β	2.33 (7.607)	0.251	4.48 (11.105)	0.698	2.33 (2.897)	0.435
IL6	3.99 (3.973)	0.649	3.99 (5.766)	0.647	4.37 (6.085)	0.373
IL8	21.34 (21.811)	0.957	17.68 (7.578)	0.490	26.56 (33.586)	0.124
IFN-α2	15.79 (12.643)	0.486	13.79 (18.549)	0.572	17.81 (8.55)	0.100
IFN-γ	4.43 (21.872)	0.124	4.87 (31.686)	0.777	4.43 (20.194)	0.244
IP-10	374.00 (29.210)	0.780	340.00 (32.485)	0.148	388.00 (56.108)	0.348
MCP1	615.00 (47.843)	0.027 *	614.00 (37.482)	0.473	615.00 (53.384)	0.486
MIP1α	7.10 (5.006)	0.955	7.10 (6.752)	0.776	7.10 (4.630)	0.837
MIP1β	64.04 (11.632)	0.850	64.04 (15.771)	0.663	63.58 (12.587)	0.900
TNFα	11.14 (3.623)	0.325	10.27 (5.314)	0.013 *	13.48 (2.543)	0.514
TNFβ	3.85 (3.769)	0.783	3.36 (5.162)	0.692	4.83 (3.986)	0.697

^†^*p*-values obtained by using Kruskal–Wallis one-way ANOVA on ranks; * Trending towards significance. No variables were statistically significant applying Bonferroni correction (*p*-value < 0.003); ** Cytokine concentrations were measured in picograms per milliliter (pg/mL).

## Data Availability

Data can be accessed by request to the corresponding author.

## Supplementary Materials

The following supporting information can be downloaded at: https://www.mdpi.com/article/10.3390/pathogens11060650/s1, Table S1 Cytokine Concentrations in Participants without Self-Reported or Clinical Depression Since WNV Infection.
