# Supplementary material for: A Preliminary Study of Proinflammatory Cytokines and Depression Following West Nile Virus Infection
Source: Pathogens. 2022 Jun 4;11(6):650. doi: 10.3390/pathogens11060650 (PMC9230011; doi:10.3390/pathogens11060650)
Supplement: Supplementary file 1 [file pathogens-11-00650-s001.zip › pathogens-1695616-supplementary.pdf]

Supplemental Table S1. Cytokine Concentrations in Participants without Self-Reported or Clinical Depression Since WNV Infection.

	No Self-Reported Depression (n=31)	No Clinical Evidence of Depression (n=21)
	median (std. error)**	median (std. error)**
G-CSF	26.47 (21.602)	32.59 (6.570)
IL12p40	35.54 (19.860)	4.66 (20.699)
IL17α	3.87 (52.855)	2.32 (14.932)
IL1α	19.30 (22.942)	29.29 (37.858)
IL1β	4.08 (4.416)	4.48 (11.105)
IL6	3.28 (7.976)	3.99 (5.766)
IL8	26.05 (34.360)	17.68 (7.578)
IFN-α2	10.56 (5.754)	13.79 (18.549)
IFN-γ	7.76 (26.127)	4.87 (31.686)
IP-10	367.50 (74.973)	340 (32.485)
MCP1	468.00 (54.250)	614 (37.482)
MIP1α	6.76 (5.655)	7.10 (6.752)
MIP1β	63.02 (15.670)	64.04 (15.771)
TNFα	13.63 (3.316)	10.27 (5.314)
TNFβ	4.67 (3.831)	3.36 (5.162)

† P-values obtained by using Kruskal Wallis one-way ANOVA on ranks

** Cytokine concentrations were measured in picograms per milliliter (pg/mL)
